# The complete chloroplast genome of *Elsholtzia fruticosa* (D. Don) Rehd. (Labiatae), an ornamental plant with high medicinal value

**DOI:** 10.1080/23802359.2023.2183069

**Published:** 2023-02-28

**Authors:** Xiao Yu, Yu-Ru Song, Zhen-Ning Zhao

**Affiliations:** a School of Landscape Architecture and Horticulture Sciences, Southwest Forestry University, Kunming, China; b School of Forestry, Southwest Forestry University, Kunming, China

**Keywords:** *Elsholtzia fruticosa*, chloroplast genome, phylogenetic analysis

## Abstract

*Elsholtzia fruticosa* is an ornamental plant with high medicinal value. In this study, we sequenced and analyzed the complete chloroplast (cp) genome of the species. The complete cp sequence is 151,550 bp, including the large single-copy (LSC) region of 82,778 bp, the small single-copy (SSC) region of 17,492 bp, and a pair of invert repeats (IRs) regions of 25,640 bp. It encodes 132 unique genes in total, including 87 protein-coding genes, 37 transfer RNA genes (tRNAs), and eight ribosomal RNA genes (rRNAs). The comparative analysis of complete cp genomes showed that the genomic structure and gene order of *E. fruticosa* cps were conserved. The sequences of *rps15*, *rps19*, *ycf1*, *ycf3*, *ycf15*, *psbL*, *psaI*, *trnG-UCC*, *trnS-GCU*, *trnR-UCU*, *trnL-UAG*, *trnP-UG*, and *trnL-UAA* serve as hotspots for developing the DNA barcoding of *Elsholtzia* species. There are 49 SSR loci in the cp genome of *E. fruticosa*, among which the repeat numbers of mononucleotide, dinucleotide, trinucleotide, tetranucleotide, and pentanucleotides SSR are 37, 9, 3, 0, and 0, respectively. A total of 50 repeats were detected, including 15 forward repeats, seven reverse repeats, 26 palindromic repeats, and two complementary repeats. Phylogenetic analysis based on the complete cp genome and protein-coding DNA sequences of 26 plants indicates that *E. fruticosa* has a dose relationship with *E. splendens* and *E. byeonsanensis*.

## Introduction

*Elsholtzia fruticosa* (D. Don) Rehd (1910) is an erect shrub belonging to the genus *Elsholtzia* of the Labiatae family (Wu 1977). Most of the plants in *Elsholtzia* are medicinal plants, some of which are edible and have broad-spectrum antibacterial and antiviral effects. *E. fruticosa* is an ornamental plant with an aromatic smell (Chen et al. 2002). It usually grows on slopes, valleys, riversides, or roadsides between 700 and 1800 m above sea level. *E. fruticosa* has high medicinal value, which can relieve rigidity of muscles and promote blood circulation (Wang and Wang 2008). The root can be used as medicine to treat rheumatism and rheumatoid arthritis pain. Leaf application can cure foot scabies. The plant’s stems, leaves, and flowers are rich in volatile oils. *E. fruticosa* has rich wild resources in southwestern China, showing potential development and application value (Kang and Shi 2009). Chloroplast (cp) genomes are widely used for phylogeny, DNA barcoding, genome evolution, and species conservation. Here, we report the complete genome of *E. fruticosa* and confirm the phylogenetic relationship, which will provide abundant information for future studies on the phylogenetic analyses and genetic diversity of *Elsholtzia* species.

## Materials and methods

### DNA extraction and sequencing, genome assembly, and annotation

**Figure 1.**
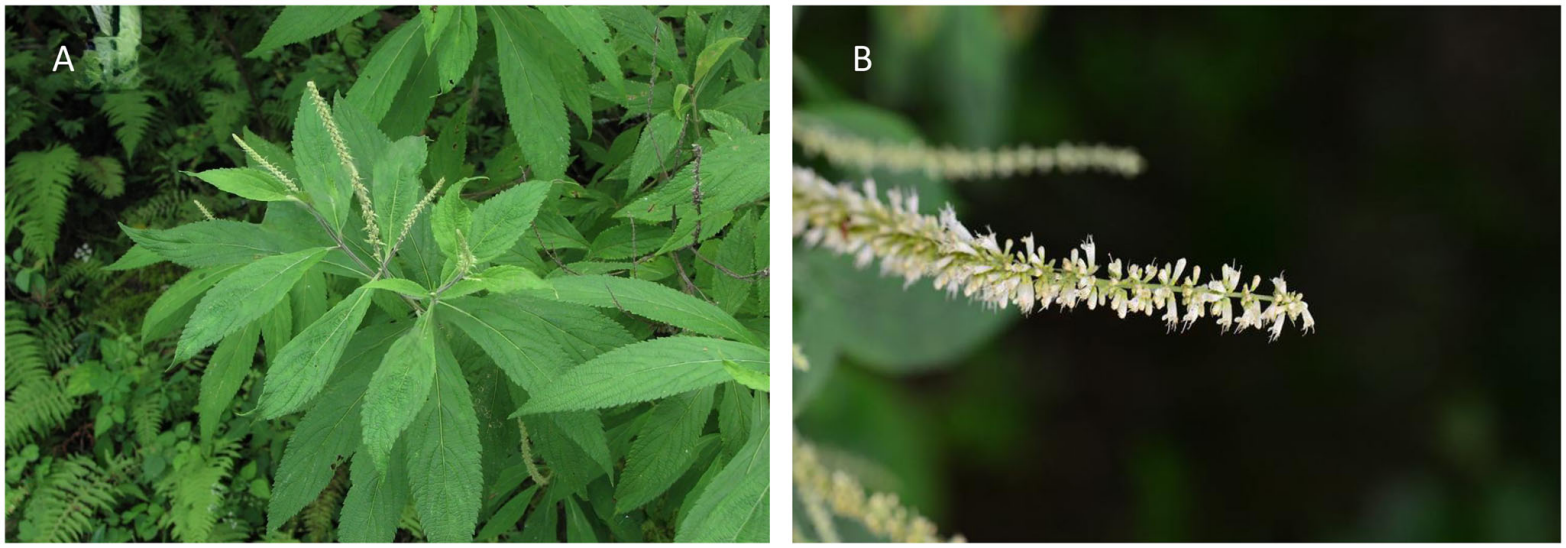
Plant morphological characteristics of *Elsholtzia fruticosa*. (A) The leaves of this species are lanceolate or elliptic lanceolate, and the base is narrow cuneate. (B) The flowers of *E. fruticosa* are cylindrical spikes, terminal or axillary. The photos of *E. fruticosa* were taken by the authors in Hutiaoxia Town, Shangri La City, Yunnan Province, China (coordinates: 99°59′33.11″E, 27°16′7.56″N).

Fresh leaves of *E. fruticosa* (Figure 1) were collected from Jinxing village, Hutiaoxia Town, Shangri La City, Yunnan Province, China (coordinates: 99°59′33.11″E, 27°16′7.56″N; altitude: 2926 m). The collection of plant materials complies with the wild plant protection regulations of the People’s Republic of China, and we obtained the permission of local authorities on forestry and the grassland bureau in Yunnan province in China. A voucher specimen (SWFU20210773MFY) was deposited in the Herbarium of Southwest Forestry University, China (http://bbg.swfu.edu.cn/, Yu Xiao, email: yuxiao0215@gmail.com). Total genome DNA was extracted with the TGuide plant genomic DNA prep kit (Tiangen Biotech, Beijing, China) from the fresh and healthy leaves of a single specimen of *E. fruticosa.* The concentration and quality of DNA were detected by agarose gel electrophoresis and spectrophotometer. The high-quality DNA was disrupted by ultrasonic waves using a Covaris instrument. After end-repairing, DNA fragment 3′ adding A, and ligation of linkers, a DNA fragment of 270 bp was selected. Polymerase chain reaction amplification was performed, and the amplified product was used to construct a library after primer-dimer removal. Finally, the qualified libraries were subjected to high-throughput sequencing on the Illumina HiSeq 4000 sequencing platform. A total of 3 G raw data from the Illumina HiSeq Platform (Illumina, San Diego, CA) were sequenced. The software Trimmomatic (Bolger et al. 2014) was employed to filter low-quality reads from the raw data and remove adapters. The complete cp genome was assembled using GetOrganelle software (Jin et al. 2018). The parameters are wordsize = 102; base coverage = 171.44; and *k* = 75, 85, 95, 105, 115, and 127 (Jin et al. 2018). This was then visualized by Bandage software (Wick et al. 2015) . The complete cp genome of *E. fruticosa* was a typical quadripartite structure (Fig. S1). The complete cp genome sequence of *E. rugulosa* (MT473758.1) was annotated using Geneious Prime (Kearse et al. 2012), and the annotations were manually corrected to obtain the final result. The OGDRAW program (https://chlorobox.mpimp-golm.mpg.de/index.html) was used to draw a detailed physical map of the *E. fruticosa* cp genome. The complete cp genome of *E. fruticosa* was submitted to GenBank with accession number ON087714.

### Analysis of tandem and simple sequence repeats (SSRs)

Simple sequence repeat analysis MISA software was used to detect the SSR loci, in which the minimum numbers of repeats for mononucleotide, dinucleotides, trinucleotides, tetranucleotides, and pentanucleotides were 10, 5, 4, 3, and 3, respectively (Hennequin et al. 2001). REPuter was used to identify long sequence repeats with the Hamming distance set at three and the minimum repeat length set at 30 bp (Kurtz et al. 2001).

### Inverted repeat contraction, expansion, and interspecific comparison

The cp genome sequence of *E. splendens* (MW900173.1), *E. rugulosa* (MT473758.1), *E. densa* (MN793319.1), and *E. byeonsanensis* (ON040655.1) was downloaded from the NCBI GenBank database. Multiple sequence alignments of entire genomes were performed using MAFFT v7.427 (Katoh and Standley 2013). Subsequently, the aligned sequences were inputted into the online tool mVISTA (http://genome.lbl.gov/vista/mvista/submit.shtml) for visual analysis of cp genome alignments (Frazer et al. 2004). The boundaries of four regions (large single-copy (LSC), small single-copy (SSC), and a pair of invert repeats (IRs)) of the cp genome were compared using the online website IRscope (https://irscope.shinyapps.io/irapp/; Amiryousefi et al. 2018). The boundary differences between IRs and SC regions among five species were analyzed by extracting IR boundary gene information.

### Phylogenetic analysis

A phylogenetic tree was reconstructed to confirm the phylogenetic location of *E. fruticosa*. At present, only the cp genome data of *E. densa*, *E. byeonsanensis*, *E. rugulosa*, and *E. splendens* have been published and are available in the NCBI database. A total of 26 plant complete cp genome sequences were used for phylogenetic reconstruction (Tab. S1), with *Thunbergia erecta* and *Barleria prionitis* as outgroups. The MAFFT v.7 program (scoring matrix = 200, PAM *k* = 2, gap open penalty = 1.53, offset value = 0.123) was used to perform multiple alignments on the cp genome sequences of these 26 plants (Katoh and Standley 2013) to test the sequence differences. Then, the comparison results were checked in Geneious 11.0.3 software and output in a *.net format file. ML tree constructed with RAxML ver.8.0.0 software, Bootstrap = 1000 (Stamatakis 2014).

At the same time, protein-coding DNA sequences (CDS) were extracted from the cp genome of each species. In order to reduce errors, the following conditions should be met when screening CDS (Wright 1990; He et al. 2016; Li et al. 2021; Tang et al. 2021): the total bases of each CDS should be an integer multiple of 3, and the sequence length should be ≥300 bp; the base types in the sequence are only A, T, C, and G; each of the sequence contains a start codon (ATG) and a stop codon (TAG, TGA, and TAA); there is no stop codon in the middle of the sequence. After removal of repetitive sequences, 53 CDS per species were retained for subsequent analysis. Chloroplast genome CDS from 26 plants were compared using MAFFT v7.0 (Katoh and Standley 2013). An ML phylogenetic tree was constructed using MEGA 7.0 software to determine bootstrap support with 1000 replicates.

## Results and discussion

### Structural characteristics

**Figure 2.**
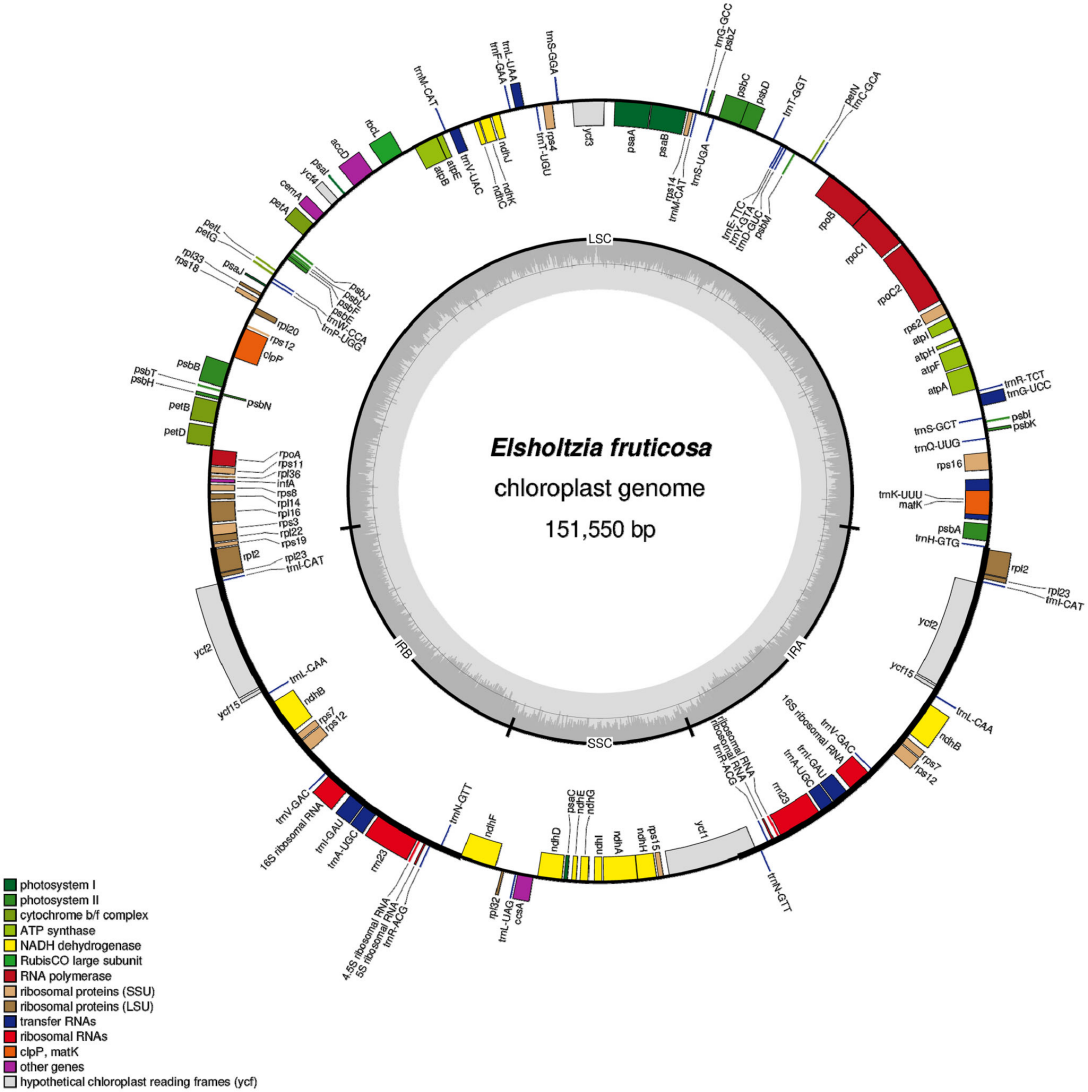
Gene map of the *Elsholtzia fruticosa* plastid genome. Genes drawn inside the circle are transcribed clockwise, and those outside are transcribed counterclockwise. Genes belonging to different functional groups are color coded. The darker gray in the inner circle corresponds to DNA G + C content, while the lighter gray corresponds to A + T content. LSC: large single-copy; SSC: small single-copy; IR: inverted repeat.

The *E. fruticosa* cp genome is a typical circular double-stranded DNA molecule with a length of 151,550 bp (Figure 2). The cp genome has the usual quadripartite structure, containing an LSC region of 82,778 bp, an SSC region of 17,492 bp, and a pair of IR regions of 25,640 bp. The base compositions of the cp genome were uneven (30.65% A, 19.32% C, 18.64% G, and 31.39% T). The overall GC and AT content of the whole genome is 37.96% and 62.04%, respectively. GC content in the IR region (43.12%) was higher than that in the LSC region (36.03%) and SSC region (32.00%). A total of 132 genes were annotated, including 87 protein-coding genes (PCGs), eight ribosomal RNA genes (rRNAs), and 37 transfer RNA genes (tRNAs). In total, 18 genes replicate in the IR region, repeating inversely with each other, including seven PCGs (*rpl2*, *rpl23*, *ycf2*, *ndhB*, *rps7*, *rps12*, *ycf15*), seven tRNA genes (*trnI-CAT*, *trnL-CAA*, *trnV-GAC*, *trnI-GAU*, *trnA-UGC*, *trnR-ACG*, *trnN-GTT*), and four rRNA genes (*rrn4.5S*, *rrn5S*, *rrn16S*, *rrn23S*). In the present study, the GC content of the *E. fruticosa* cp genome sequence was consistent with most of the reported dicotyledon cp GC content ranges (31–38%). The higher GC content in the IR region may be due to the presence of rRNA genes, while the SSC region contains most of the NADH genes (Cai et al. 2006).

### Sequence repeats analysis

A total of 49 SSRs were discovered by the online software MISA-web (Beier et al. 2017). Among them, the numbers of mono-, di-, tri-, tetra-, and pentanucleotides SSRs are 37, 9, 3, 0, and 0, respectively. A total of 50 repeats were identified in the cp genome of *E. fruticosa*, including 15 forward repeats, seven reverse repeats, 26 palindromic repeats, and two complementary repeats. Those with lengths of 20–30 bp accounted for the majority of repetitive sequences (68%). These results provide a good resource for SSR makers for the study of genetic diversity and species identification between *E. fruticosa* and related species.

### Inverted repeat contraction, expansion, and interspecific comparison

By comparing the boundaries between the IR region and the LSC and SSC regions of the cp genomes of the five *Elsholtzia* species, it was found that the cp genomes of *Elsholtzia* species were relatively conserved, and there were some differences in the cp genome boundaries of the five plants (Fig. S2). The longest cp genome was *E. rugulosa* (151,952 bp), and *E. densa* (149,095 bp) was the shortest. The difference in genome length mainly occurred in the LSC region. The IR region of five *Elsholtzia* species was longer, and the SSC region was shorter, indicating that the IR region of the cp genome might expand during the evolution of *Elsholtzia*. The *ndhF* genes of five *Elsholtzia* species spanned IRb and SSC, and the *ycf1* genes spanned SSC and IRa. Except for *E. densa*, the *rps19* genes of the other four plants crossed the LSC and IRb boundaries.

In this study, the cp genome of *E. rugulosa* was used as a reference for mVISTA to compare the cp genomes of five *Elsholtzia* species (Fig. S3). MVISTA-based identification maps revealed DNA sequence conservation and gene synchronization with the complete cp genome, revealing regions of increased genetic variation. The results showed that the genome composition and size of the five species were quite similar. These species show a closer relationship because they belong to the same genus. However, some differences remain in the cp genome sequences of *Elsholtzia* species. The differences are noncoding regions > coding regions and LSC > SSC > IRs. The sequences with significant differences in coding regions included *rps15*, *rps19*, *ycf1*, *ycf3*, *ycf15*, *psbL*, and *psaI*, and the sequences with significant differences in noncoding regions included *trnG-UCC*, *trnS-GCU*, *trnR-UCU*, *trnL-UAG*, *trnP-UGG*, and *trnL-UAA*. These highly variable sequences are expected to be reliable molecular markers for *Elsholtzia* species in future studies.

### Phylogenetic analyses

**Figure 3.**
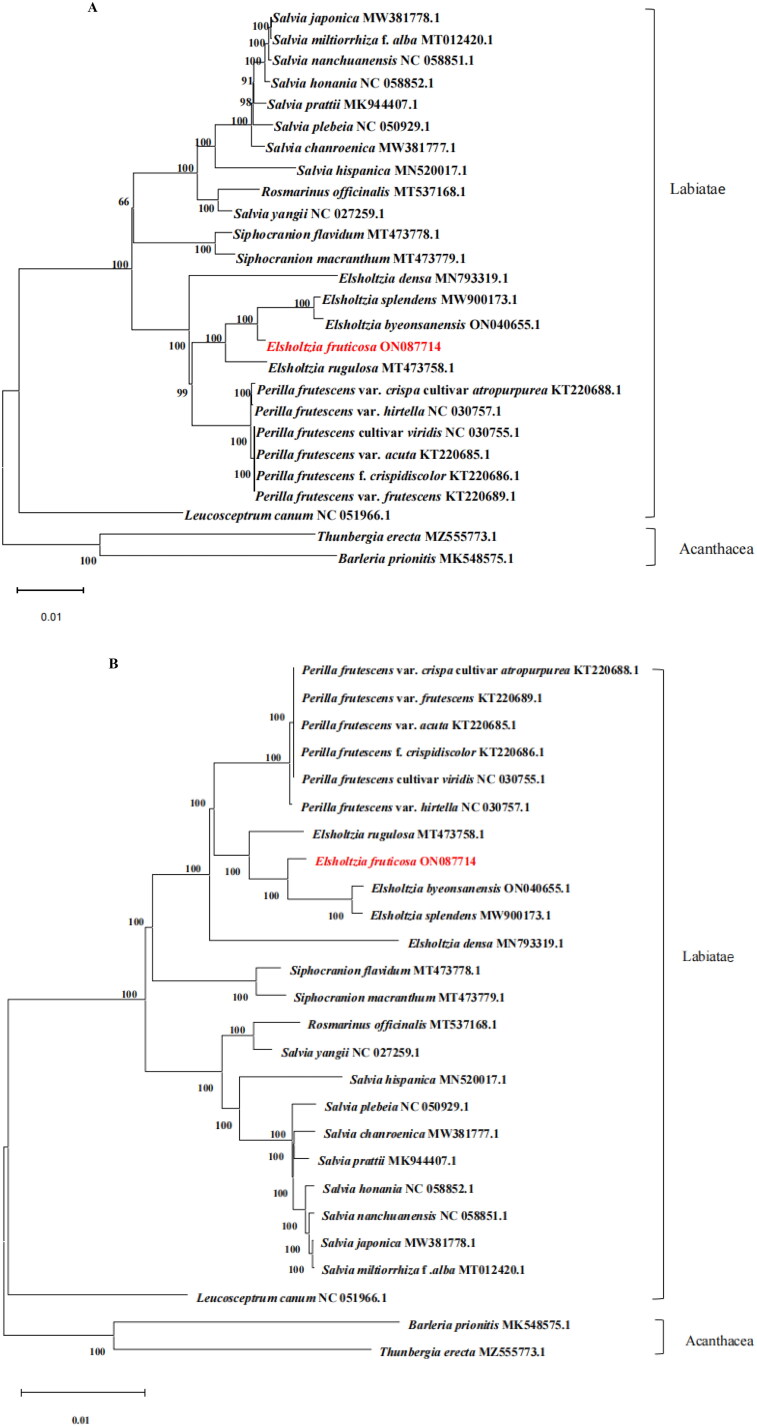
The best maximum-likelihood (ML) phylogenetic tree reconstructed by RAxML ver. 8.0.0 based on complete chloroplast genome (A) and the chloroplast protein-coding DNA sequences (B) of 24 species of Labiatae, including five *Elsholtzia* species, *Thunbergia erecta* and *Barleria prionitis* as outgroups. The numbers on branches are bootstrap support values from 1000 replicates. The following sequences were used: *Salvia japonica* MW381778.1 (Li et al. 2021), *Salvia miltiorrhiza* f. *alba* MT012420.1 (Lin et al. 2022), *Salvia nanchuanensis* NC 058851.1 (Li et al. 2021), *Salvia honania* NC 058852.1 (Wang et al. 2022), *Salvia prattii* MK944407.1 (Xia et al. 2022), *Salvia plebeia* NC 050929.1 (Paje et al. 2022), *Salvia chanroenica* MW381777.1 (Cui et al. 2021), *Rosmarinus officinalis* NC 027259.1 (Gonçalves et al. 2022), *Salvia yangii* MT537168.1 (Kozłowska et al. 2022), *Siphocranion flavidum* MT473778.1 (Chen et al. 2019), *Siphocranion macranthum* MT473779.1 (Feng et al. 2021), *Elsholtzia densa* MN793319.1 (Liang et al. 2021), *Elsholtzia splendens* MW900173.1 (Huang et al. 2022), *Elsholtzia byeonsanensis* ON040655.1 (Chen et al. 2022), *Elsholtzia rugulosa* MT473758.1 (Liu et al. 2022), *Perilla frutescens* var. *crispa* cultivar *atropurpurea* KT220688.1 (Salachna et al. 2021), *Perilla frutescens* var. *hirtella* NC 030757.1 (Salachna et al. 2021), *Perilla frutescens* cultivar *viridis* NC 030755.1 (Salachna et al. 2021), *Perilla frutescens* var. *acuta* KT220685.1 (Salachna et al. 2021), *Perilla frutescens* f. *crispidiscolor* KT220686.1 (Salachna et al. 2021), *Perilla frutescens* var. *frutescens* KT220689.1 (Salachna et al. 2021), *Leucosceptrum canum* NC 051966.1 (Yan et al. 2022), *Thunbergia erecta* MZ555773.1 (Zakaria et al. 2022), and *Barleria prionitis* MK548575.1 (Comito et al. 2022).

Phylogenetic trees constructed based on the complete cp genome (Figure 3(A)) and the cp protein-coding DNA sequences (Figure 3(B)) have very high similarity, 24 Labiatae species and two Acanthaceae species were supported as a monophyletic lineage. *Elsholtzia* is closely related to *Perilla*. Our result indicated that *E. fruticosa* has a closer relationship with *E. splendens* and *E. byeonsanensis*, with 100% bootstrap support (Figure 3(A,B)). The phylogenetic results are also consistent with the results of the tribal classification of Lamiaceae based on plastome phylogenomics (Zhao et al. 2021). However, due to the limited sequence of *Elsholtzia* cp genomes, the phylogenetic relationship in this family requires further study. Therefore, to further study the evolutionary history of *E. fruticosa*, a more complete *Elsholtzia* species cp sequence is required.

This study used Illumina sequencing data for the first time to examine the complete cp genome of *E. fruticosa*. The unveiling of the cp genome sequence of *E. fruticosa* will provide significant information for the phylogeny and plant molecular identification of *Elsholtzia* species.

## Supplementary Material

Supplemental Material

## Data Availability

The genome sequence data that support the findings of this study are openly available in GenBank of NCBI at https://www.ncbi.nlm.nih.gov under the accession no. ON087714. The associated BioProject, SRA, and Bio-Sample numbers are PRJNA820615, SRR18500750, and SAMN27019135, respectively.
