# Supplementary material for: The complete chloroplast genome of Elsholtzia fruticosa (D. Don) Rehd. (Labiatae), an ornamental plant with high medicinal value
Source: Mitochondrial DNA B Resour. 2023 Feb 28;8(3):336–41. doi: 10.1080/23802359.2023.2183069 (PMC9980026; doi:10.1080/23802359.2023.2183069)
Supplement: Supplemental Material [file TMDN_A_2183069_SM7457.docx]

**Content：**

**Table S1.** Complete chloroplast genome information used in the phylogeny of this study

**Fig S1.** The coverage figure of the complete chloroplast genome of *E. fruticosa*.

**Fig S2.** Comparison of the borders of the LSC, SSC, and IR regions of five *Elsholtzia* species

**Fig S3.** Comparison of five *Elsholtzia* species chloroplast genomes using *E. rugulosa* as a reference sequence with a 50% identity cutoff. Gray arrows show the position and direction of each gene. The colored areas indicate the exon, intron, and intergenic spacer (IGS) sequences. The vertical axis indicates the sequence.

**Table S1**. Complete chloroplast genome information used in the phylogeny of this study

| Family | Genus | Species | GenBank accession number | Length |
| --- | --- | --- | --- | --- |
| Acanthaceae | *Elsholtzia* | *Elsholtzia splendens* Nakai | MW900173.1 | 151224 bp |
| Acanthaceae | *Elsholtzia* | *Elsholtzia rugulosa* Hemsl | MT473758.1 | 151962 bp |
| Acanthaceae | *Elsholtzia* | *Elsholtzia densa* Benth. | MN793319.1 | 149095 bp |
| Acanthaceae | *Elsholtzia* | *Elsholtzia byeonsanensis* M.Kim | ON040655.1 | 150628 bp |
| Acanthaceae | *Salvia* | *Salvia japonica* Thunb. | MW381778.1 | 151394 bp |
| Acanthaceae | *Salvia* | *Salvia miltiorrhiza* f. *alba* C.Y.Wu et H.W.Li | MT012420.1 | 151389 bp |
| Acanthaceae | *Salvia* | *Salvia nanchuanensis* Sun | NC 058851.1 | 151568 bp |
| Acanthaceae | *Salvia* | *Salvia honania* L. H. Bailey | NC 058852.1 | 151559 bp |
| Acanthaceae | *Salvia* | *Salvia prattii* Hemsl. | MK944407.1 | 151690 bp |
| Acanthaceae | *Salvia* | *Salvia plebeia* R. Br. | NC 050929.1 | 151062 bp |
| Acanthaceae | *Salvia* | *Salvia chanroenica* Nakai | MW381777.1 | 151647 bp |
| Acanthaceae | *Salvia* | *Salvia hispanica* Ettling. ex Willk. & Lange | MN520017.1 | 150980 bp |
| Acanthaceae | *Salvia* | *Salvia yangii* B. T. Drew | NC 027259.1 | 152462 bp |
| Acanthaceae | *Rosmarinus* | *Rosmarinus officinalis* L. | MT537168.1 | 151473 bp |
| Acanthaceae | *Siphocranion* | *Siphocranion flavidum* Y. P. Chen & C. L. Xiang | MT473778.1 | 152039 bp |
| Acanthaceae | *Siphocranion* | *Siphocranion macranthum* (Hook. f.) C. Y. Wu | MT473779.1 | 152126 bp |
| Acanthaceae | *Perilla* | *Perilla frutescens* var. *crispa* cultivar *atropurpurea* (Thunb.) Hand.-Mazz. | KT220688.1 | 152598 bp |
| Acanthaceae | *Perilla* | *Perilla frutescens* var. *hirtella* (Nakai) Makino | NC 030757.1 | 152656 bp |
| Acanthaceae | *Perilla* | *Perilla frutescens* cultivar *viridis* (L.) Britt. | NC 030755.1 | 152588 bp |
| Acanthaceae | *Perilla* | *Perilla frutescens* var. *acuta* (L.) Britt. | KT220685.1 | 152588 bp |
| Acanthaceae | *Perilla* | *Perilla frutescens* f. *crispidiscolor* Makino | KT220686.1 | 152598 bp |
| Acanthaceae | *Perilla* | *Perilla frutescens* var. *frutescens* (Thunb.)Hand.-Mazz | KT220689.1 | 152598 bp |
| Acanthaceae | *Leucosceptrum* | *Leucosceptrum canum* Smith | NC 051966.1 | 152739 bp |
| Acanthacea | *Thunbergia* | *Thunbergia erecta* (Benth.) T. Anders | MZ555773.1 | 152202 bp |
| Acanthacea | *Barleria* | *Barleria prionitis* L. | MK548575.1 | 152217 bp |


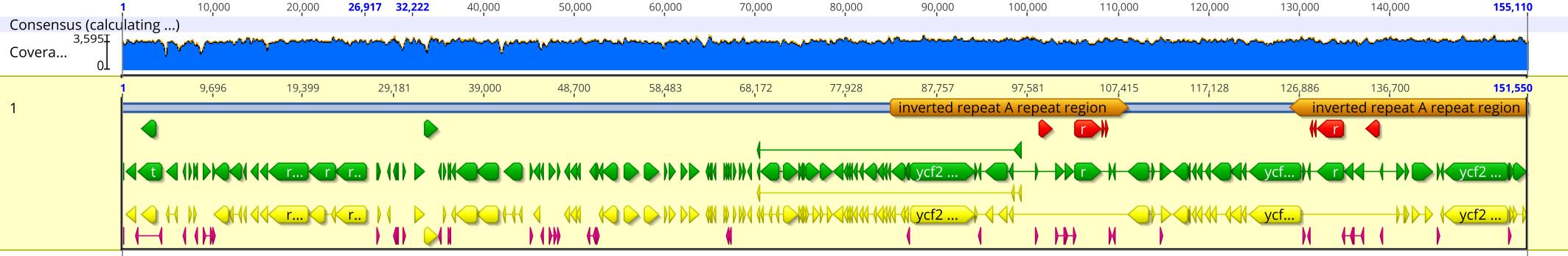


**Fig S1.** The coverage figure of the complete chloroplast genome of *E. fruticosa*. This figure was generated using Geneious Prime software by aligning sequencing data with the assembled sequence. The upper region shows the coverage of each locus in the complete chloroplast genome of *E. fruticosa*. The lower region shows the genome length, gene location, and tetrad region information of the genome. Through the figure, each locus of the complete chloroplast genome can be arranged by reads. It can be used as evidence that the genome is circular.


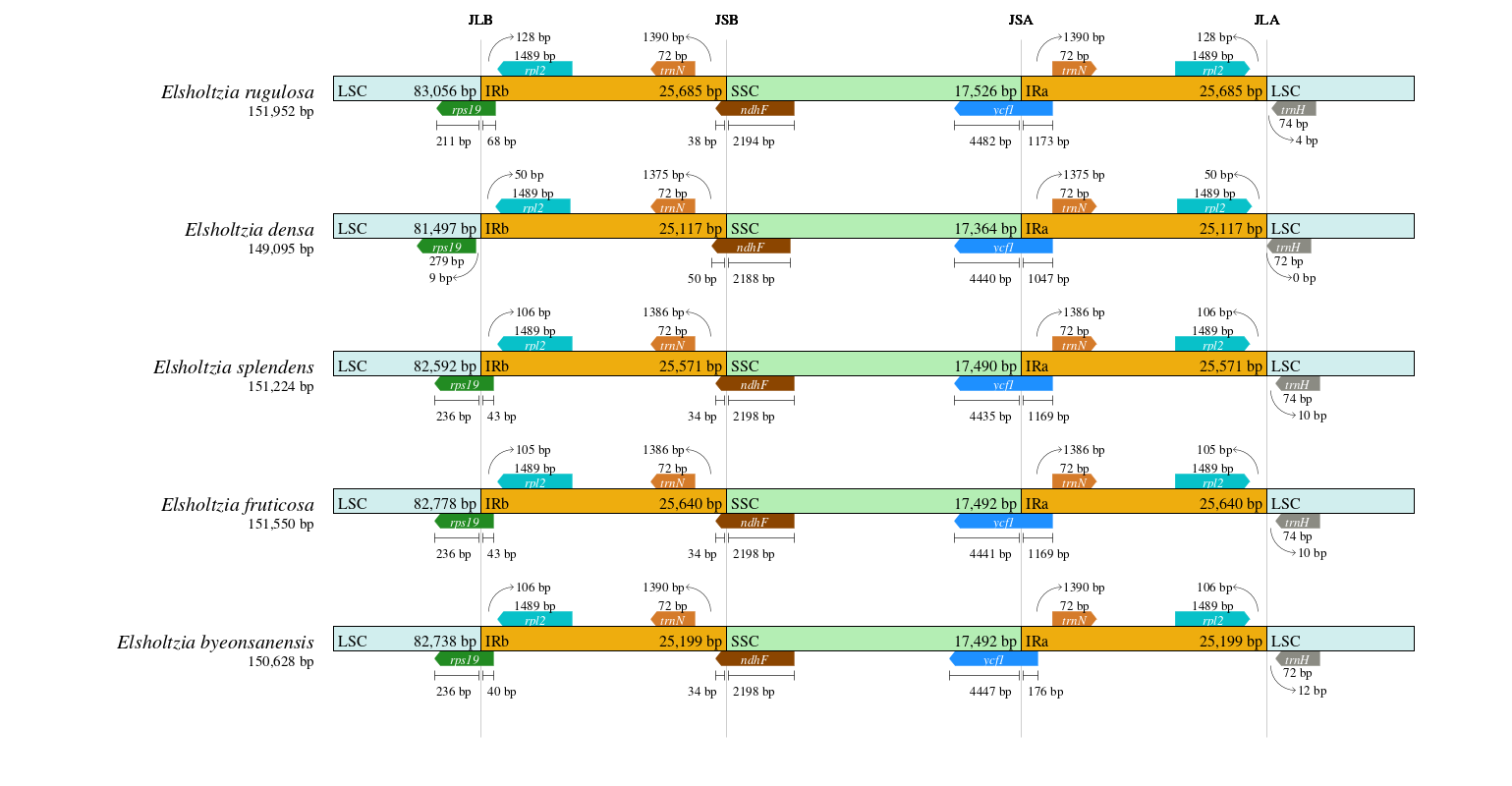


**Fig S2** Comparison of the borders of the LSC, SSC, and IR regions of five *Elsholtzia* species


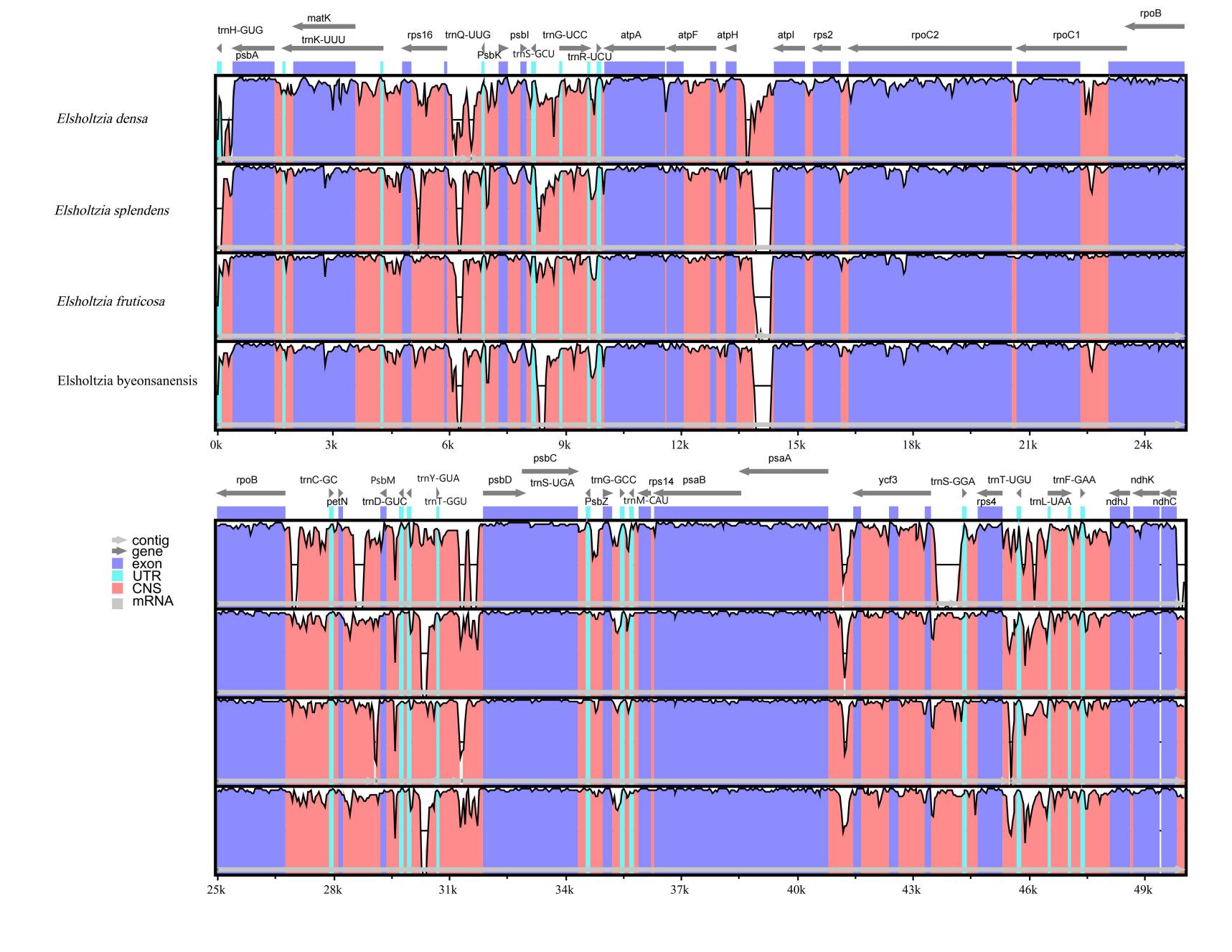

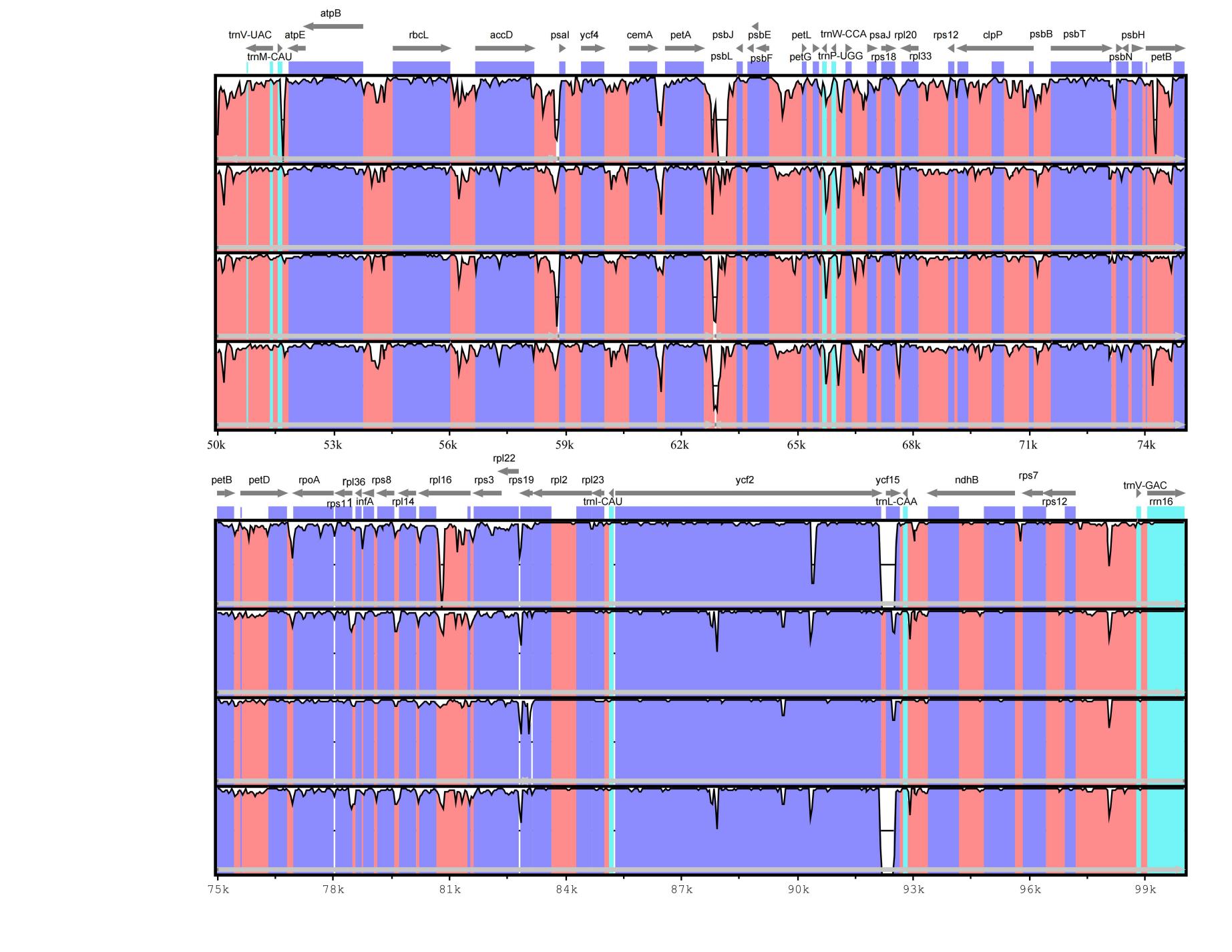

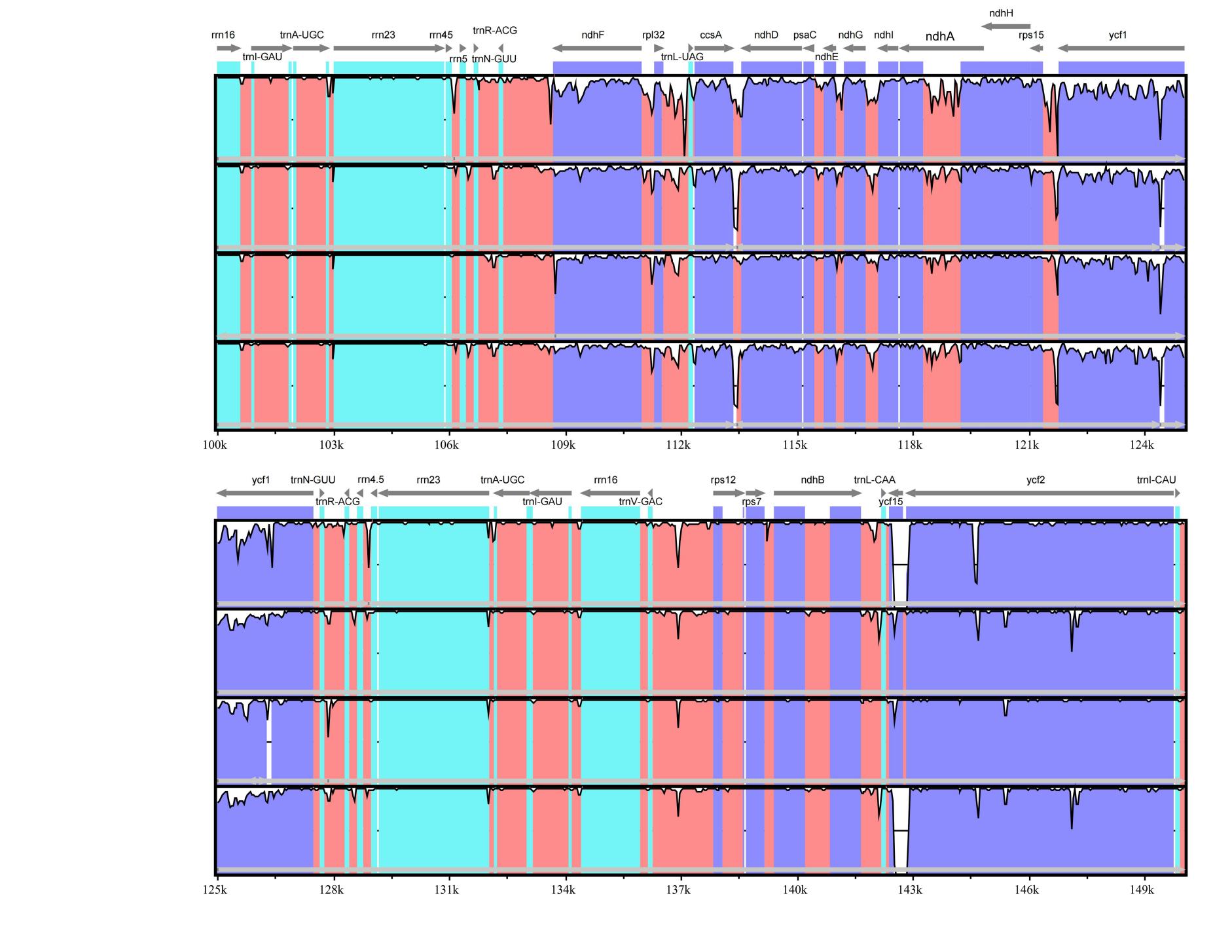


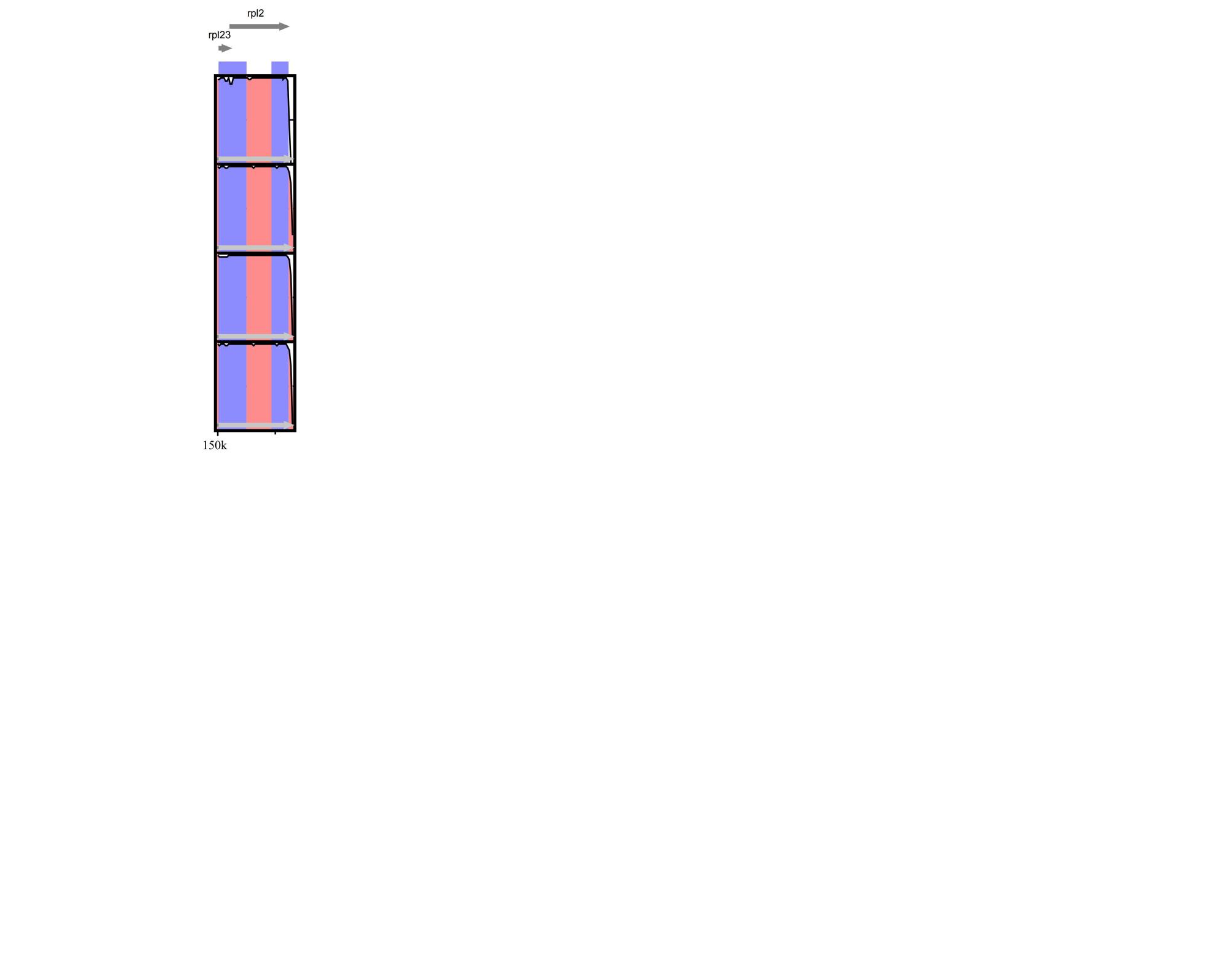


**Fig S3** Comparison of five *Elsholtzia* species chloroplast genomes using *E. rugulosa* as a reference sequence with a 50% identity cutoff. Gray arrows show the position and direction of each gene. The colored areas indicate the exon, intron, and intergenic spacer (IGS) sequences. The vertical axis indicates the sequence.
